# Paralysis Following Measles

**Published:** 1894-01

**Authors:** 


PARALYSIS FOLLOWING MEASLES. (*Gazette des Hopitaux*, September 14 and 19, 1893.) By P. A. Lop, M. D.

Paralysis following measles is a comparatively rare affection, less than a hundred cases having been reported at all. This is probably due, however, to its usual transitory charactor and to the fact that it appears most frequently in the third week of convalescence—after the patients have passed from immediate observation. Two adult cases have been reported, but the usual time of its appearance is that of the appearance of other forms of paralysis.

In the cerebral form, which is most grave but less frequent, paralysis occurs preceded by spasmodic muscular movements and exaggerated reflexes. The electrical reactions remain normal, however, ana the disease runs a rapid course.

In the spinal variety, on the contrary, the reflexes are rapidly lost, and the electric reactions are much modified, but the course is most frequently benign. It usually takes the form of paraplegia, commmencing with formications and cramps, followed by retention of urine and incontinence of fæces. Occasionally it is of the ascendant variety ending in death by paralysis of the diaphragm. Its diagnosis is easy, as it occurs in convalescence from a fever; its course is rapid, from one to six weeks; the spinal form is frequent, and the patient always recovers.

Its pathology is, probably, a congestion caused by a specific bacillary toxine in the blood, which in the grave forms has produced a true inflammation and atrophic sclerosis.—*International Medical Mag.*

